# Analysis of 22-year Drought Characteristics in Heilongjiang Province Based on Temperature Vegetation Drought Index

**DOI:** 10.1155/2022/1003243

**Published:** 2022-04-28

**Authors:** Li Wu, Youzhi Zhang, Limin Wang, Wenhuan Xie, Lijuan Song, Haifeng Zhang, Hongwen Bi, Yanyan Zheng, Yu Zhang, Xiaofei Zhang, Yan Li, Zhiqun Lv

**Affiliations:** ^1^Institute of Agricultural Remote Sensing and Information, Heilongjiang Academy of Agricultural Sciences, Haerbin 150086, China; ^2^Institute of Agricultural Regional Chinese Academy of Agricultural Sciences, Beijing 100081, China

## Abstract

Heilongjiang Province is the main grain producing region in China and an important part of Northeast China Plain, which is one of the three black soil belts in the world. The cultivated region of black soil accounts for 50.6% of the black soil region in Northeast China. Due to the obvious rise of temperature and uneven distribution of precipitation in the 20th century, it has been considered to be one of the important reasons for agricultural drought and aridity. Under the background of climate change, understanding the multiyear changes and occurrence characteristics of cultivated land drought in different agricultural regions in Heilongjiang Province is of great significance for the establishment of agricultural drought prediction and early warning system in the future, guiding agricultural high-standard farmland irrigation in different regions, promoting black soil protection, and then improving grain yield. This paper calculates the temperature vegetation drought index (TVDI) based on the normalized difference vegetation index (NDVI) and surface temperature (TS) product data of MODIS from 2000 to 2021. Taking TVDI as the drought evaluation index, this paper studies the temporal and spatial variation distribution characteristics and occurrence frequency of drought in the whole region and four agricultural regions of Heilongjiang Province: Daxing an Mountain and Xiaoxing an Mountain (region I), Sanjiang Plain (region II), Zhangguangcai Mountains (region III), and Songnen Plain (region IV). The results show that medium drought generally occurred in Heilongjiang Province from 2000 to 2021, accounting for about 70% of the total cultivated land. The drought was severe from 2000 to 2009 and weakened from 2010 to 2021. In the 110 months of the crop growing season from 2000 to 2021, about 63.84% of the region suffered more than 60 droughts. It is found that the frequency of drought varies from region to region. More than 80 droughts occurred in the west of region IV and the middle of region II. The characteristics of region IV are large sandstorm, less precipitation, and lack of water conservancy facilities, resulting in frequent and strong drought. It is also found that the occurrence frequency, degree grade and regional distribution of drought are closely related to seasonal changes. In spring, the occurrence grade and frequency of drought in region IV are the strongest and the drought phenomenon is serious. In autumn, drought is frequent and distributed in all regions, but the grade is not strong (mainly medium drought), and the drought phenomenon is medium. It is humid in summer. Crops in Heilongjiang Province are one crop per annual. Spring drought seriously restricts the water content of crops. Long-term drought will lead to poor crop development and reduce yield. Therefore, only by clarifying the characteristics of regional time drought, monitoring accurate drought events and accurately predicting the occurrence of drought, can we guide high-standard farmland precision irrigation, improve crop yield and ensure national food security. At the same time, severe drought will affect the terrestrial ecosystem, resulting in the distribution of crops and microorganisms, and the transformation between carbon sink and carbon source.

## 1. Introduction

Heilongjiang Province is the main grain producing region in China and an important part of Northeast China Plain, which is one of the three black soil belts in the world. It is an important commodity grain base in China, known as “Beidacang”. The western part of black soil region is located in the dual-transition zone of climate and ecology. It is a climate change-sensitive region with serious desertification [1]. Heilongjiang Province has become one of the main warming regions in China since 1980, and the average temperature has increased by 1.4℃ in recent 100 years (1905–2001). Drought has become one of the major natural disasters in the province. If the arid area and drought intensity level cannot be accurately determined or effective countermeasures cannot be taken, farmers' agricultural income will suffer serious losses by 2030 [2]. Drought is affected by many factors, such as climate warming, soil erosion, land use/land cover change, and human activities. In recent years, black land degradation and protection, drought monitoring, and early warning have always been the focus of research. The eighth phase strategic plan (2014–2021) of the international hydrological plan (IHP) takes the change of water-related disasters affected by global climate change and intense human activities as one of the key research issues and coordinates the research on drought through the international drought initiative (IDI) to improve the ability to cope with drought [3].

According to the statistics, from 2000 to 2019, the drought-affected region of crops in Heilongjiang Province has reached 2.1374 million hectares every year [4]. In the past 20 years, the drought area accounted for 63% of the total area of meteorological disasters, ranking the first in the province. Under the background of global climate change, extreme weather and drought events occur from time to time, such as the severe drought in the summer of 2000 [5], in which the drought-affected area of the whole province reached 5.016 million hectares and the drought time reached 30 to 40 days, and in 2002, the temperature in the whole province was generally on the high side, the precipitation in the northwest was less, and the drought situation was severe [6]. The research focus of this paper is to monitor and analyze the temporal and spatial variation characteristics and frequency of drought by using remote sensing indicators. Previous studies mainly used meteorological drought indicators and historical statistical data to monitor drought. The monitoring mainly analyzed the point results of meteorological stations or transformed the points into surface results through analysis methods. The accuracy of the results was limited by the number of stations and analysis methods. Using remote sensing indicators to monitor the spatial-temporal and frequency characteristics of drought in Heilongjiang Province was rare. The previous research took Heilongjiang Province as the research area, did not subdivide the region of the province, and the research on the temporal, spatial, and frequency characteristics of drought in different agricultural regions of Heilongjiang Province was also rare. Most research results [7–9] did not clearly define the occurrence, development, and end period of drought. It is very important to monitor and predict the occurrence and development of drought in the whole region and different agricultural regions of Heilongjiang Province, and accurately evaluate it. It is very important to carry out precision irrigation according to the different levels of drought in different regions at different times. Drought is a common natural disaster, which is gradual. Its occurrence and end are slow and difficult to detect. For drought-prone areas, accurate quantitative expression of drought is very important for effective drought management. Remote sensing drought monitoring indicators can be divided into two categories: one is the monitoring of a single index, including a single vegetation index or a single temperature index, and the other is the monitoring of a double index, considering both temperature and vegetation index. Commonly used single indexes include anomaly vegetation index (AVI), temperature state index (TCI), and normalized difference vegetation index (NDVI). However, the research shows that [10], the factors affecting drought are complex, and it is limited to use a single index to monitor drought. The double indexes include temperature vegetation drought index (TVDI), conditional vegetation index (VTCI), and vegetation water supply index (VSWI). Both vegetation index and temperature are considered, so drought can be retrieved effectively. Yu Min [11] used TVDI to monitor the summer drought in Heilongjiang Province in 2007, showing that this method can monitor the drought in real time and truly reflect the dynamic process of local drought occurrence and development. Zhong Wei [12] analyzed the characteristics of soil moisture in Lhasa based on TVDI and found that TVDI has a good negative correlation with the measured surface soil moisture. Using TVDI, it is monitored that the arid area is mainly distributed in the north, southwest, and some parts of central and southern China.

This paper first compares the TVDI method of remote sensing drought index with the standard precipitation evapotranspiration index (SPEI) method of mature meteorological drought index and clarify the advanced nature and superiority of TVDI method. Second, this paper monitors the drought change situation of 110 months in the whole growth season of Heilongjiang Province in 22 years (every May to September in 2000–2021), and analyzes the spatial distribution characteristics, occurrence frequency, and future trend of Heilongjiang drought. This study is conducive to accurately grasp the multiyear changes and occurrence characteristics of drought in the whole region and agricultural subregion of Heilongjiang Province. It can not only establish an effective drought monitoring [13–15] and early warning system to guide the accurate and effective irrigation of high-standard farmland but also explore the response mechanism of ecological environment and surface crops to climate change and provide reference for agricultural production and managers [16].

## 2. Data and Methods

### 2.1. Overview of the Study Region

**Figure 1:**
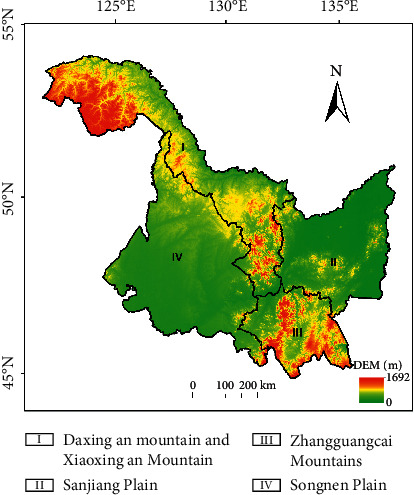
Scope of study region and partition.

As the largest commercial grain production base in China, Heilongjiang Province is facing severe challenges in regional drought prevention and disaster reduction due to the impact of climate warming and uneven rainfall distribution. Heilongjiang Province (121°11′–135°05 ′E, 43°25′–53 °33′N) is located in Northeast China. It is the province with the highest latitude in China. The terrain is characterized by two mountains in the north and south and two plains in the east and west (Figure 1). The crops in the study region are one crop per annual, and the whole growth period is from May to September, with an annual average temperature of −6°C to 4°C in the region. According to the temperature index from south to north, it can be divided into middle temperate zone and cold temperate zone. The annual precipitation is 350 to 650 mm. From east to west, it can be divided into humid region, semihumid region, and semiarid region according to the dryness index [17]. Agricultural characteristic area in Heilongjiang Province is divided into four regions [18] (Figure 1): Daxing an Mountain and Xiaoxing an Mountain (region I), Sanjiang Plain (region II), Zhangguangcai Mountains (region III), and Songnen Plain (region IV). Region I is mainly forestry and a small amount of agriculture, mainly wheat, soybean, and potato. Regions II and IV are mainly agriculture, mainly planting corn, rice, soybean, wheat, potato, and so on. Region III is dominated by agriculture and forestry, and agriculture is mainly planted with rice, corn, soybean, and so on.

### 2.2. Data Processing

#### 2.2.1. MODIS\Terra NDVI and Ts Products

Remote sensing MODIS products (2640 scenes) covering all the study regions: vegetation index (MOD13A2) and surface temperature (MOD11A2) data. MODIS sensors transit twice a day, which can achieve full coverage of the research region. The data come from the computer network information center of the Chinese Academy of Sciences (https://www.nsdata.cn/). The collection time of MOD11A2 product (global 1 km 8-day surface surface temperature/emissivity data) is from day 121 to day 273 every year (2000–2021), and the collection time of MOD13A2 product (global 1 km 16-day vegetation index data) is the same. The maximum value method is used to unify the product time of MOD13A2 and MOD11A2 to 16 days.

#### 2.2.2. Acquisition of Cultivated Land Information

The MODIS Collection 5.1 land use/land cover product data set (MCD12Q1), the University of Boston in the United States, is used for the study of cultivated land information, which is sourced from NASA website (https://ladsweb.nascom.nasa.gov/data/search.html). The spatial resolution in 2012 was 500 meters. MCD12Q1 data have five data layers, and the overall classification accuracy ranges within 74.8% ± 1.3%. The main types include forest land, grassland, cultivated land, wetland, and urban construction land. In this paper, the selection of cultivated land is the phase element with DN value of 12 in the first classified image [19].

#### 2.2.3. Meteorological Data Observed by Meteorological Stations

**Figure 2:**
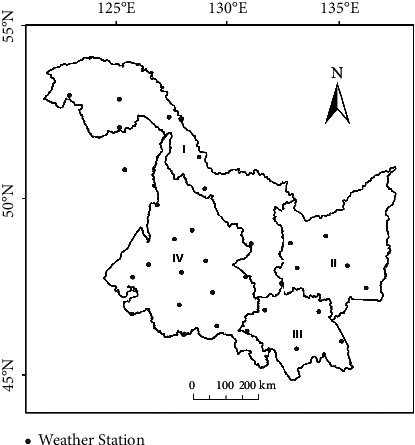
Distribution of meteorological stations.

The distribution of meteorological stations in Heilongjiang Province is shown in Figure 2. The meteorological data (2000–2019) includes the monthly (May to September) average temperature and precipitation data monitored by each station, which are derived from China meteorological data sharing network (https://cdc.nmic.cn/). There are nearly 6000 valid data.

### 2.3. Research Methods

#### 2.3.1. TVDI Method

**Figure 3:**
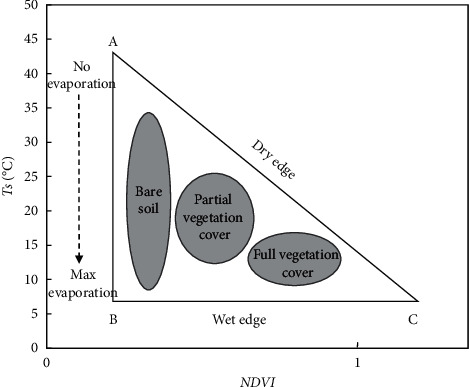
NDVI-Ts triangular feature space.

*2.3.1.1 Spatial Characteristics of NDVI-Ts*: Through analysis, Goetz [20] considered that there is an obvious negative correlation between NDVI and Ts. The main reason is that the underlying surface temperature will rise sharply when the vegetation is subjected to water stress. He estimated the regional average soil humidity conditions and considered that the resolution of the sensor has little effect on the relationship between them. Price et al. [21] analyzed the NDVI and Ts data obtained by different satellite sensors and considered that the scatter diagram composed of NDVI and surface radiation temperature was triangular (Figure 3). The spatial distribution of soil moisture in the region can be obtained by obtaining the regional vegetation index and surface temperature from satellite data, establishing the scatter map of them, and determining the coordinates of dry edge, wet edge, and each vertex of the model. In this paper, the monthly scale Ts-NDVI space is established by using monthly remote sensing data. The maximum and minimum Ts corresponding to each NDVI value are the maximum and minimum combination methods respectively. The maximum Ts value defines the dry edge and the minimum Ts value defines the wet edge. The calculation is as follows:

(1) $$\begin{array}{c} Ts_{\max}=a_{1}+b_{1}\times NDVI, \end{array}$$

(2) $$\begin{array}{c} Ts_{\min}=a_{2}+b_{2}\times NDVI. \end{array}$$

In this formula, *Ts* is the surface temperature; *Ts*_min_ is the minimum surface temperature under the same NDVI conditions; *Ts*_max_ is the maximum surface temperature under the same NDVI conditions. *a*_1_, *a*_2_, *b*_1_, and *b*_2_ are the coefficients of the fitting equation.

**Figure 4:**
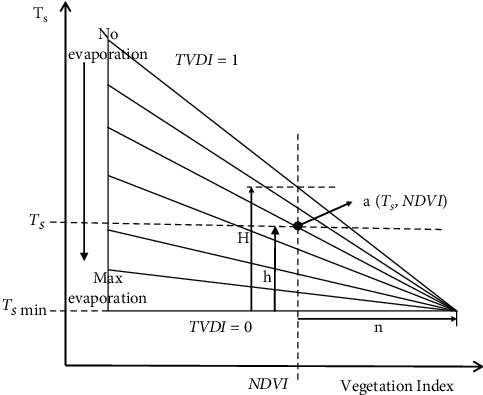
TVDI model principle.

*2.3.1.2 TVDI Establishment*: TVDI is a drought monitoring model proposed by Sandholt et al. [22] based on the characteristic space of NDVI-Ts. Its principle is that NDVI-Ts characteristic space has a series of soil moisture and other straight lines. These isolines are the slopes of Ts and NDVI under different water conditions. Therefore, the concept of TVDI is proposed (Figure 4). The calculation formula is as follows:

(3) $$\begin{array}{c} TV\;DI=\frac{Ts-Ts_{\min}}{Ts_{\max}-Ts_{\min}}. \end{array}$$

In this formula, *Ts*, *Ts*_min_, and *Ts*_max_ are the same as formulas (1) and (2).

Substitute formulas (1) and (2) into formula (3), that is,

(4) $$\begin{array}{c} TV\;DI=\frac{Ts-\left(a_{2}+b_{2}\times N\;DV\;I\right)}{\left(a_{1}+b_{1}\times N\;DV\;I\right)-\left(a_{2}+b_{2}\times N\;DV\;I\right)}. \end{array}$$

In this formula, *Ts*, *a*_1_, *a*_2_, *b*_1_, and *b*_2_ are the same as formulas (1) and (2).

*2.3.1.3 TVDI Classification*: The grade division of TVDI can characterize the degree of drought, and the TVDI value is between 0 and 1. According to the author's previous research, the TVDI drought grade standard suitable for Heilongjiang Province is: 0 < TVDI < 0.46 is normal, 0.46 ≤ TVDI < 0.57 is light drought, 0.57 ≤ TVDI < 0.76 is medium drought, 0.76 ≤ TVDI < 0.86 is severe drought, and 0.86 ≤ TVDI < 1 is extreme drought [23]. In order to facilitate the drawing, this paper expands the TVDI value by 100 times for analysis, that is, the TVDI value is between 0 and 100 (Table 1).

#### 2.3.2. Standardized Precipitation Evapotranspiration Index (SPEI) Method

The standardized precipitation evapotranspiration index (SPEI) characterizes the drought condition of a region according to the degree that the difference between precipitation and evapotranspiration deviates from the average state. SPEI is constructed by considering the impact of temperature on drought and introducing potential evapotranspiration based on SPI index. Therefore, SPEI is also an index based on probability model and also has the characteristics of multitime scale. The specific calculation steps are shown by Li [24]. This paper only lists the main steps: (1) calculate the potential evapotranspiration (PET) by using Penman–Monteith formula; (2) calculate the difference *D* between monthly precipitation and potential evapotranspiration; (3) the cumulative probability density of *D* was calculated by three parameter log-logistic; and (4) normalized *P* = 1 − *F*(*x*)

When cumulative probability *p* ≤ 0.5, $\text{W}=\sqrt{-2\;\ln\text{P}}$

(5) $$\begin{array}{c} \text{SPEI}=\omega -\frac{c_{0}+c_{1}\omega +c_{2}\omega^{2}}{1+d_{1}\omega +d_{2}\omega^{2}+d_{3}\omega^{3}}. \end{array}$$

When cumulative probability *P* > 0.5: $\text{W}=\sqrt{-2\;\ln\left(1-\text{P}\right)}$

(6) $$\begin{array}{c} \text{SPEI}=-\left(\omega -\frac{c_{0}+c_{1}\omega +c_{2}\omega^{2}}{1+d_{1}\omega +d_{2}\omega^{2}+d_{3}\omega^{3}}\right). \end{array}$$

In the formula, *c*_0_ = 2.5155, *c*_1_ = 0.0103, *d*_1_ = 1.4328, *d*_2_ = 0.1893, *d*_3_ = 0.0013, and *P* is precipitation.

SPEI drought classification of TVDI is shown as Table 2.

#### 2.3.3. Drought Spatial Distribution Analysis Method

*2.3.3.1 TVDI Average Method*: In this study, the average value method (formula (7)) is used to analyze the spatial pattern distribution characteristics of drought, TVDI_22_ is obtained, and it is graded and marked according to the drought grade, so as to obtain the average spatial distribution of drought in Heilongjiang Province in 22 years.

(7) $$\begin{array}{c} {\text{TVDI}}_{22}=\frac{\left({\text{TVDI}}_{2000}+{\text{TVDI}}_{2001}+\cdots {\text{TVDI}}_{2021}\right)}{22}. \end{array}$$

In this formula, TVDI_22_ represents the 22-year average value of each TVDI pixel within Heilongjiang Province, TVDI_2000_, TVDI_2001_… TVDI_2021_ represents the annual average value of each TVDI pixel within Heilongjiang Province from 2000 to 2021.

*2.3.3.2 TVDI Range Method*: The range value represents the difference between the maximum value and the minimum value of each TVDI pixel in Heilongjiang Province in the past 22 years (formula (8)). The greater the range value, the greater the interannual drought gap, indicating that the pixel is vulnerable to drought years. The smaller the range value, the smaller the interannual drought gap, indicating that it is not affected by drought years and is in the same state all year round.

(8) $$\begin{array}{c} {\text{TVDI}}_{R}={\text{TVDI}}_{\max}-{\text{TVDI}}_{\min}. \end{array}$$

In this formula, TVDI_R_ represents the interannual range of each TVDI pixel in Heilongjiang Province, TVDI_max_ is the maximum value of each TVDI pixel in 22 years, and TVDI_min_ is the minimum value of each TVDI pixel in 22 years.

#### 2.3.4. Drought Assessment Method

*2.3.4.1 Calculation of region Proportion of Drought Grade*: The proportion of drought grade region represents the scope of drought in the study region. The formula is expressed as follows:

(9) $$\begin{array}{c} P_{i}=\frac{m}{M}\times 100\text{\%.} \end{array}$$

In this formula, *i* is the drought grade, representing normal, light, medium, severe, and extreme drought; *m* is the number of pixels with *i* drought level; and *M* is the total number of pixels of all drought levels.

*2.3.4.2 Calculation of Drought Frequency*: In this paper, TVDI ≥ 57 is defined as the standard for a certain degree of drought [25], which stipulates that if TVDI_i_ ≥ 57, it is considered that a certain degree of drought occurs, and the given frequency value is 1; otherwise, TVDI_i_ < 57, it is considered that a certain degree of drought does not occur, and the given frequency value is 0. TVDI_i_ is the TVDI of the *i* month of the year (May to September), and the total drought frequency is the sum of the occurrence frequency of each month.

## 3. Results and Analysis

### 3.1. Comparison between TVDI Method and SPEI Method

**Figure 5:**
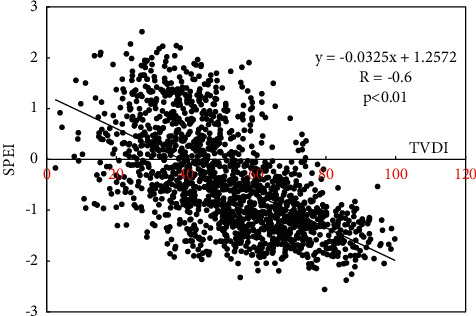
Relationship analysis between monthly SPEI and monthly TVDI.

The standardized precipitation evapotranspiration index (SPEI) is one of the meteorological drought indexes. The calculation of SPEI in this paper is based on the impact of precipitation, temperature, and evapotranspiration items monitored by the meteorological station (Figure 2) on meteorological drought, which can well describe the meteorological drought. Many scholars [26–31] have shown that this index is an ideal tool for monitoring crop drought. However, the deficiency of this method is that the monitoring results are meteorological station values and point data. Relying only on the data of meteorological stations cannot fully reflect the drought characteristics of the whole province. This is also the advantage of remote sensing monitoring drought, which can realize the full coverage of the province and provide area data. Graphs from remote sensing are fundamental models used in scientific approaches to describe the relation between objects in the real world [32]. Scholars use professional meteorological data interpolation software to spatially interpolate points and obtain surface data [33]. Owing to the limitations of the number of meteorological stations and conversion methods, the accuracy of monitoring surface drought is also limited. Owing to the complexity of region, environment and climate, TVDI has more advantages in monitoring drought pixel by pixel. Many scholars have found that the drought monitoring method of TVDI is superior and more feasible [34–38]. In this paper, the TVDI value and SPEI value of meteorological stations are selected to correspond one-to-one, and the correlation between them is analyzed (Figure 5). It is found that there is a negative correlation between January scale SPEI and monthly TVDI. The formula is SPEI = −0.0325TVDI + 1.2572, and the correlation coefficient is −0.6, *P* < 0.01. It shows that there is a very significant negative correlation between monthly TVDI value and monthly scale SPEI value, which shows that using TVDI to monitor drought can not only monitor large-scale regional drought in the whole province, but also retain the monitoring accuracy of SPEI method. The retrieval data of vegetation index and ground temperature obtained by remote sensing satellite can well express the ground agricultural drought.

### 3.2. Analysis of Spatial Characteristics of Drought in Heilongjiang Province from 2000 to 2021

**Figure 6:**
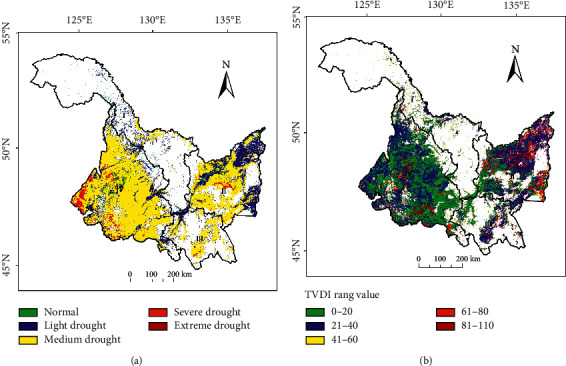
Results of average drought and range difference of the whole province in 22 years: (a) TVDI_22_ drought grade distribution map and (b) TVDI_R_ map.

The spatial distribution results of average TVDI (Figure 6(a)) and TVDI range value (Figure 6(b)) from 2000 to 2021 show that the annual average TVDI and range value TVDI can reflect the occurrence, severity, and interannual changes of drought in Heilongjiang Province. Spatially, there are few cultivated lands in region I and are rarely affected by drought, and there is little difference between the middle ages of drought in region III. Regions IV and II carry the main cultivated land resources in the province. Region IV shows the level of perennial drought, especially in the west. There are various drought levels in region II. The drought in the east is the lightest and rarely affected by drought, but the difference between ages is obvious (TVDI_R_ > 60). There are a small number of perennial severe drought regions in the middle, and the drought in other areas is medium. Region II is the region with the largest difference in drought change between ages.

### 3.3. Analysis of Drought Time Characteristics in Heilongjiang Province from 2000 to 2021

#### 3.3.1. Annual Characteristic Analysis

**Figure 7:**
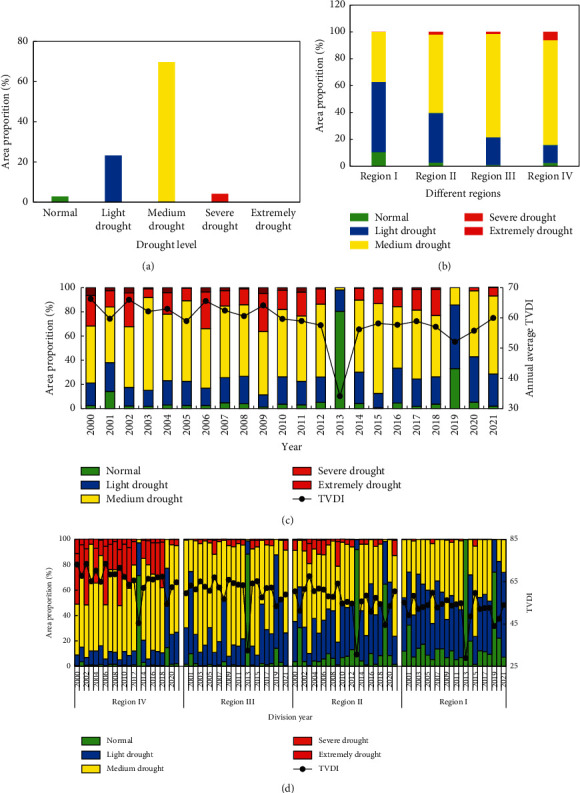
Characteristics of annual drought change in province and regions: (a) Proportion of drought grade region in the whole province. (b) Proportion of drought grade region in different regions. (c) Characteristics of annual drought change in province. (d) Characteristics of annual drought change in regions.

The proportion of average TVDI drought grade area in Heilongjiang Province from 2000 to 2021 (Figure 7(a)) shows that the medium drought grade region is the largest, accounting for 70% of the total region, the light drought region accounts for 23%, the severe drought region accounts for 4%, the normal region accounts for 3%, and the extreme drought area accounts for 0. The area above medium drought level accounts for 74%, indicating that drought is widespread in Heilongjiang Province, mainly in medium drought level, and drought is serious in some regions.

The proportion of the average TVDI drought grade area in the four agricultural regions of Heilongjiang Province from 2000 to 2021 (Figure 7(b)) shows that the proportion of the sum of normal and light drought regions is 63% in region I, 40% in region II, 22% in region III, 16% in region IV. The proportion of severe drought area in region IV is the largest among the four regions, accounting for 6%. Therefore, the drought-affected levels of the four regions are region I, region II, region III, and region IV from low to high.

The annual average TVDI and the proportion of drought grade region in Heilongjiang Province from 2000 to 2021 (Figure 7(c)) show that the annual average TVDI from 2000 to 2009 is high, and the sum of severe drought and severe drought areas was 32%, 16%, 32%, 8%, 22%, 11%, 34%, 15%, 14%, and 36%, with an average of 22%, indicating that the drought in Heilongjiang Province from 2000 to 2009 was severe, and there was a major drought every other year. The severest years are 2009, 2006, 2002 and 2000 respectively, which are dry years. After 2010, the average TVDI decreased, and the proportion of severe drought and extreme drought was 18%, 23%, 14%, 0%, 10%, 13%, 16%, 18%, 23%, 0%, 3%, and 7%, with an average of 12%, indicating that the drought after 2010 was weaker than before, especially in 2013 and 2019.

The annual average TVDI and the proportion of drought grade region in the four agricultural regions of Heilongjiang Province from 2000 to 2021 (Figure 7(d)) show that the annual average TVDI in region IV is consistent with the annual average TVDI (7C) of the whole province. Except for a few years (2003, 2013, 2019, 2020, and 2021), the proportion of severe drought and extreme drought areas are large, especially in the four dry years, it reaches 50%. The average is 25% in 22 years. Therefore, it shows that region IV in the province is affected by drought with large area and strong grade, which is a typical arid area. In region III, the proportion of severe drought and extreme drought is 3% on average, and the proportion of normal and light drought areas is 33% on average. In 22 years, the proportion of area with severe drought and extreme drought in region II is 6% on average, and the proportion of area with normal and light drought is 46% on average. The sum of area with severe drought, extreme drought and area with normal and light drought in region III is smaller than that in region II. The results are consistent with the analysis in Figure 7(b). It shows that the drought-affected area in region III is large, but the level is not strong, mainly medium drought. The drought levels in region II are diverse, and there are certain areas with severe drought and extreme drought. The proportion of normal and light drought area in region I is 60% on average, and it is humid all year round.

#### 3.3.2. Monthly Characteristics Analysis

**Figure 8:**
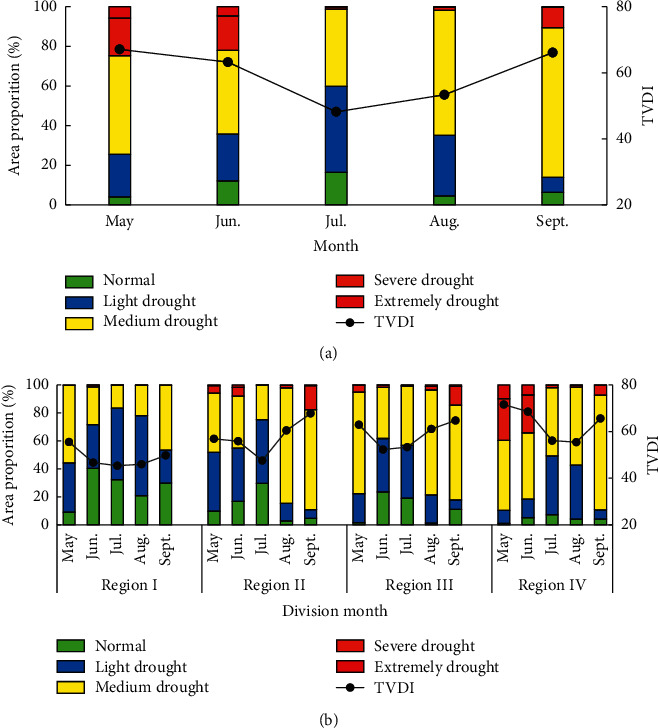
Characteristics of monthly drought change in province and regions: (a) characteristics of monthly drought change in province and (b) characteristics of monthly drought change in regions.

The proportion of monthly average TVDI and drought grade area in Heilongjiang Province from 2000 to 2021 (Figure 8(a)) shows that the average value of annual monthly TVDI is 48 in July and 53 in August, belonging to light drought. It is 67 in May, 63 in June and 66 in September, belonging to medium drought. In May, the area of severe drought and extreme drought accounted for 25%, and the area of normal and light drought accounted for 26%. In June, the proportion of severe drought and extreme drought decreased to 22%, and the proportion of normal and light drought increased to 36%, indicating that the area of drought (aforementioned medium drought) in June decreased and the grade weakened compared with that in May. In September, the proportion of severe drought and extreme drought areas decreased to 11%, and the proportion of normal and light drought areas decreased to 14%, indicating that the drought area in September is large, and the drought grade is concentrated, mainly at the level of medium drought. Crops in Heilongjiang province belong to the one crop per annual. Drought in spring (May) and early summer (June) affects crop growth and development. Severe drought will reduce crop yield. Autumn (September) is the mature season. A certain degree of drought is conducive to crop maturity and harvest.

The proportion of monthly average TVDI and drought grade area in the four agricultural regions in the province from 2000 to 2021 (Figure 8(b)) shows that the average TVDI values in region I from May to September are 55.50, 46.68, 45.36, 45.98, and 49.78, of which, there is no drought in July and August and light drought in May, June, and September; Region II: 56.88, 55.87, 47.58, 60.48, and 67.78, of which May, June, and July are light drought and August and September are medium drought; Region III: 62.94, 52.3, 53.31, 61.09, and 64.75, of which May, August, and September are medium drought and June and July are light drought; Region IV: 71.59, 68.52, 56.1, 5.46, and 65.6, of which May, June, and September are medium drought and July and August are light drought. The change trend of TVDI in region IV from May to September is the same as that in the whole province from May to September. The proportion of severe drought and extreme drought in region IV in May is the largest in all months and regions, accounting for 40%, followed by 34% in June. The results show that the areas with large drought area and serious grade in the crop growth season in Heilongjiang Province are spring (May) and early summer (June) in region IV, which will hinder the germination and growth of crops.

To sum up, the research on the temporal and spatial changes of Heilongjiang Province and four agricultural regions shows that TVDI can monitor the occurrence and development of large-scale drought and judge its drought degree, but it cannot directly express the frequency of drought.

### 3.4. Analysis of Drought Frequency in Heilongjiang Province

**Figure 9:**
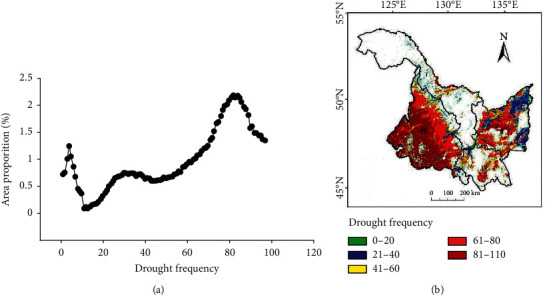
Total frequency of drought in Heilongjiang Province from 2000 to 2021 A and proportion of different frequency regions B: (a) total frequency of drought and (b) proportion of different frequency regions.

Analyze the area proportion of the total frequency of drought in Heilongjiang Province from 2000 to 2021 (Figure 9(a)) and the regional distribution of different frequencies (Figure 9(b)). The drought frequency distribution is similar to that of TVDI_22_. Surface temperature and effective precipitation determine the spatial distribution of drought grade and drought frequency. Figure 9(b) shows that the areas with drought frequency less than 40 times are mainly distributed in the east of region I and region II, and the areas with drought frequency more than 81 times are mainly distributed in the west of region IV and the middle of region II. In the 110 months during the crop growing season (May to September) from 2000 to 2021, about 22.28% of the cultivated land in the province has a drought frequency of less than 40 times, about 13.88% of the cultivated land has a drought frequency of 40 to 60 times, and about 63.84% of the cultivated land has a drought frequency of more than 60 times.

**Figure 10:**
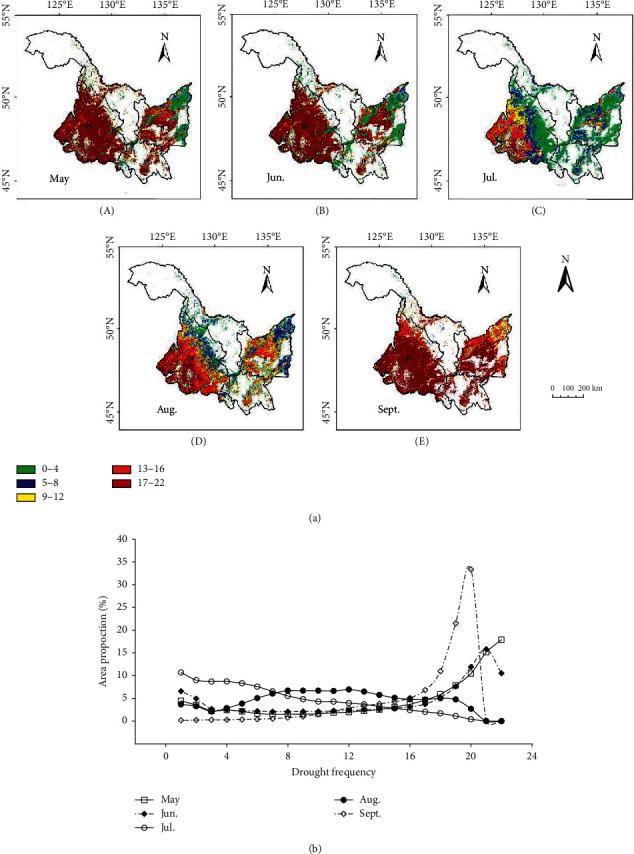
(a) Spatial distribution of drought frequency in Heilongjiang Province from May to September 2000–2021. (b) Proportion of drought frequency region in Heilongjiang Province from May to September 2000–2021.

Analyze the distribution of drought occurrence frequency (Figure 10(a)) and the proportion of drought area in Heilongjiang Province (Figure 10(b)) accumulated from May to September in the past 22 years. The results show that the average monthly drought frequency in the province is 15.71 times in May, 14.49 times in June, 7.18 times in July, 10.92 times in August and 17.34 times in September respectively. The highest drought occurrence frequency in the province is in September and May, respectively. The area with 22 droughts accounted for 17.9% in May, and the area with 20 droughts accounted for 33.4% in September. The area with one drought accounted for 10.7% in July and 0.2% in September. The area in May and September with less than 16 droughts accounted for no more than 5%.

By analyzing the drought frequency of four agricultural regions accumulated in 22 years from May to September, it is found that the average drought frequency from May to September in region I is 11.3 times, 9.1 times, 3.6 times, 5.5 times, and 12.5 times, respectively, that in region II is 11.6 times, 10.4 times, 5.4 times, 9.4 times, and 16.2 times, that in region III is 13.8 times, 10 times, 5 times, 9 times, and 18.4 times, and that in region IV is 18.5 times, 17.5 times, 8.6 times, 12.5 times, and 18.2 times. The highest frequency of drought in each region is in May and September of region IV and September of region III. The local rainfall in spring (May) and autumn (September) is less, and it is more vulnerable to strong winds, accelerating soil water evaporation and aggravating the frequency of drought.

Drought occurred frequently and widely from 2000 to 2021, which basically ran through the whole crop growth season, but there were some differences in drought area, drought frequency, and drought degree. The drought in spring and autumn is serious, frequent, and affects a wide area. Although the drought in summer is not serious, it is frequent and common in the southwest of region IV.

In different years, in the beginning of rainy season, there is a certain randomness in the amount of rainfall and the distribution of rainfall. There is also a certain seasonality of rainfall on the regional scale. Therefore, drought itself has a certain randomness and periodicity. Drought frequency is an appropriate index to describe the randomness and periodicity of drought. The drought frequency in the whole province decreases from spring to summer and then increases from late summer to autumn. This paper finds that the drought frequency in the whole growing season of crops in regions I, II, and III changes to some extent, while the drought frequency in the whole growing season of crops in the southwest of region IV has been very high, indicating that precipitation in Heilongjiang province plays a significant role in the formation of a drought cycle. Precipitation is mainly concentrated in summer, but less in the southwest of region IV.

## 4. Discussion

Using the surface TVDI data covering the whole province, this paper analyzes the temporal and spatial characteristics and frequency of drought at different time and spatial scales, and reveals the occurrence, development, severity, and change law of drought in Heilongjiang Province in the past 22 years. Li Chongrui [33] used SPEI to analyze the drought law of corn in the time scale (1, 3, 6, 12, and 24 months) from 1989 to 2018 in Northeast China, and made it clear that the drought was more serious from 2000 to 2010, the high incidence month of drought was May, with the largest drought area and degree, and the southwest of Heilongjiang Province was a high incidence area of drought. This result is consistent with the results of this paper. This paper analyzes the temporal and spatial characteristics of drought from four agricultural regions. The planting structure in each region is different. This paper does not discuss and analyze different crop types in different regions, and the data analysis is limited by the age of remote sensing data. In the future, while continuously improving and supplementing remote sensing data, we will continue to study artificial intelligence and machine learning methods [39, 40], which can extract different crop types on the ground and monitor the response of different crop types to drought. If we can analyze the damage degree of early and late maturing crops in different drought periods and regions, and guide the research of suitable crop varieties in different regions according to the analysis results, it will be very meaningful.

## 5. Conclusion

From 2000 to 2021, the drought phenomenon of cultivated land in Heilongjiang Province was frequent and serious. In the whole crop growing season, the medium drought degree was the most prominent, accounting for 70% of the total cultivated land, which was distributed throughout the province. In the 110 months of monitoring, the drought frequency was more than 80 times, mainly distributed in the west of region IV and the middle of region II, and the drought frequency of about 63.84% of the cultivated land occurred more than 60 times. During the 22 years of TVDI monitoring, the drought was serious from 2000 to 2009, which was alleviated after 2010.2000, 2002, 2006, and 2009 were dry years, and 2013 and 2019 were wet years. Although the climate temperature in Heilongjiang Province has gradually increased in recent years, the short-term drought has been alleviated due to the influence of strong rainfall. Among the four agricultural regions in the province, region IV is a typical arid area, which is subject to large drought area and heavy intensity all year round, especially in May and June every year. The drought area is the largest but medium intensity in September. At the same time, the drought frequency in May and September is also the most. The drought levels in region II are diverse, with strong drought in the middle and weak drought in the east. Its diverse drought levels lead to large interannual fluctuations and are more vulnerable to drought years. The crop in Heilongjiang province belongs to the once crop per annual. September is the autumn harvest season. The mild drought is conducive to the maturity and harvest of crops. There is less rainfall in spring and is easy to be affected by strong winds, which intensifies the occurrence of drought. The strong drought in spring and early summer (May-June) seriously affects the germination and growth of crops. The total frequency distribution of annual drought is similar to that of TVDI_22_, and there is a significant correlation between TVDI and SPEI, indicating that precipitation and surface temperature determine the spatial distribution of drought grade and drought frequency. Owing to the uncertainty of rainfall time, rainfall, and distribution, drought is also accompanied by randomness and periodicity. Therefore, regional drought must be accurately monitored, and its drought occurrence mechanism also needs more in-depth discussion. It will be more meaningful to consider social, human, natural, and other influencing factors.

## Figures and Tables

**Table 1:** Drought classification of TVDI in Heilongjiang Province.

Normal	Light drought	Medium drought	Severe drought	Extreme drought
(0, 246)	[46, 57)	[57, 76)	[76, 86)	[86, 100)

**Table 2:** SPEI drought classification.

Normal	Light drought	Medium drought	Severe drought	Extreme drought
(−0.5, +*∞*)	(−1.0, −0.5]	(−1.5, −1.0]	(−2.0, −1.5]	(−*∞*, −2.0]

## Data Availability

The data used to support the findings of this study are available from the corresponding author upon request.
